# Symptomatic intracranial hemorrhage mediates the association between eosinophils and 90-day outcomes after mechanical thrombectomy for acute ischemic stroke

**DOI:** 10.1186/s12868-023-00820-5

**Published:** 2023-12-08

**Authors:** Shuhong Yu, Xiaocui Wang, Hao Huang, Yi Luo, Zhiliang Guo

**Affiliations:** 1 Department of Encephalopathy, Suzhou Integrated Traditional Chinese and Western Medicine Hospital, Suzhou, 215101 China; 2 Department of Neurology and Suzhou Clinical Research Center of Neurological Disease, The Second Affiliated Hospital of Soochow University, No.1055, Sanxiang Road, Suzhou, 215004 Jiangsu China

**Keywords:** Eosinophils, Symptomatic intracranial hemorrhage (sICH), Outcome, Neuroprotection, Mechanical thrombectomy.

## Abstract

**Background:**

Decreased eosinophil level is associated with poor outcomes after mechanical thrombectomy in patients with acute ischemic stroke (AIS), but the underlying mechanism of this association is elusive. We aimed to assess the mediation effect of symptomatic intracranial hemorrhage (sICH) on the aforementioned association.

**Methods:**

A total of 328 consecutive AIS patients undergoing mechanical thrombectomy between May 2017 and March 2021 were analyzed. SICH was defined as any evidence of brain hemorrhage on CT scan with neurological deterioration. Regression analysis was used to assess the effect of eosinophils on sICH, and its effect on poor outcome. Mediation analysis was performed to assess the proportion of total effect by sICH on the association between eosinophils and poor outcome.

**Results:**

Multivariate analysis revealed an independent association between eosinophil count and sICH after adjusting for potential confounders (odds ratio, 0.00; 95% CI, 0.00–0.01; *P* = 0.0141), which is consistent with the result of eosinophil count (dichotomous) as a categorical variable (odds ratio, 0.22; 95% CI, 0.11–0.46; *P <* 0.0001). Eosinophil count was negatively associated with poor outcome (odds ratio, 0.00; 95% CI, 0.00–0.02; *P* = 0.0021). Mediation analysis revealed that sICH partially mediated the negative relationship between eosinophil count and poor outcome (indirect effect=-0.1896; 95%CI: -0.3654 – -0.03, *P* < 0.001).

**Conclusion:**

This study showed an important effect of sICH on the association between eosinophils and poor outcome.

**Supplementary Information:**

The online version contains supplementary material available at 10.1186/s12868-023-00820-5.

## Background

Reduced eosinophil levels are associated with poor prognosis after mechanical thrombectomy in patients with acute ischemic stroke (AIS), but the underlying mechanism of this association is unclear [1, 2]. In contrast, in our previous study, lower eosinophil levels were associated with a higher incidence of stroke-associated pneumonia (SAP); in addition, SAP was found to play an important mediating role in the association between eosinophilia and poor prognosis. Furthermore, on multivariate analysis, eosinophils showed a significant association with poor outcomes independent of SAP [1]. These results suggest that eosinophils may contribute to poor prognosis through other pathways besides SAP, such as cerebral ischemia-reperfusion injury and subsequent symptomatic intracranial hemorrhage (sICH).

Eosinophils can secrete a variety of chemokines and vascular endothelial growth factors [3–5]. Chemokines secreted by eosinophils can induce activation of M2 phenotype microglia and have a neuroprotective effect by promoting the resolution of inflammation. Moreover, vascular endothelial growth factor may be neuroprotective by regulating angiogenesis [3–5]. Therefore, it is plausible that the decrease in eosinophils aggravates cerebral ischemia-reperfusion injury, which in turn leads to sICH and subsequent poor outcomes. From this perspective, an increase in sICH may possibly mediate the association between eosinophilia and poor outcomes.

In the present study, we aimed to assess whether, in addition to SAP, sICH also has an important mediating role in the association between eosinophilia and poor outcomes. Our results may provide new insights to unravel the mechanisms of cerebral ischemia-reperfusion injury and sICH.

## Materials and methods

### Study population

Consecutive patients with AIS who received endovascular treatment at the Department of Neurology at our institution between December 2017 and March 2021 were prospectively recruited. Selection criteria for patients receiving first-line therapy, including direct aspiration, stent retriever, or a combination of stent retriever and local aspiration catheter, have been previously published [1]. To retrospectively analyze the relationship between eosinophils, sICH, and functional outcomes, the exclusion criteria were: (1) patients receiving intra-arterial thrombolytic therapy only (n = 56); (2) asthma, eosinophilic esophagitis, hypereosinophilic syndrome, evidence of active infection, chronic inflammation, autoimmune disease, steroid therapy, cancer, hematologic disease, or severe liver and kidney dysfunction (n = 4); and (3) unavailability of complete blood counts, medical records, or loss to follow-up (n = 9). A total of 328 patients with AIS met the study selection criteria and were included in the analysis (participant selection flowchart: Supplementary Fig. 1). The study protocol was approved by the Ethics Committee of the Second Hospital of Soochow University, and written informed consent was obtained from all patients or their relatives.

### Clinical protocol and laboratory tests

Baseline characteristics including demographics (age, sex), potential stroke risk factors (atrial fibrillation, hypertension, diabetes, hyperlipidemia, history of smoking and alcohol consumption), stroke etiology, stroke severity, and Alberta Stroke Program Early CT Score (ASPECTS) at admission were collected. Intravenous thrombolysis pretreatment (IVT) on admission, pre-morbid modified Rankin Scale (mRS) score, site of occlusion, the status of collateral circulation, time to reperfusion at symptom onset or last seen, and blood index were also retrieved. Risk factors for stroke were defined according to previously published criteria [6]. Etiological subtypes and stroke severity were recorded as previously described [1, 7]. The status of collateral circulation prior to thrombectomy was assessed using the American Society of Interventional and Therapeutic Neuroradiology/Association of Interventional Radiology scale [8]. At the final angiogram, reperfusion status was graded according to the modified Thrombolysis in Cerebral Infarction (mTICI) score, with successful reperfusion defined as a score of 2b or 3 [1, 7]. Peripheral venous blood samples were obtained on admission to measure eosinophil levels. Patients were followed up by telephone or outpatient visits for 3 months. A modified Rankin Scale score of 3–6 at month 3 after admission was considered poor outcome [1, 7].

### Evaluation of intracranial hemorrhage

On admission, all patients underwent CT scans. CT examinations were repeated immediately after mechanical thrombectomy and at 24 to 48 h, thereafter; in case of rapid neurological deterioration, another CT scan was performed immediately to assess the presence of sICH. CT images were reviewed by a neuroradiologist with extensive experience in acute stroke, keeping medical records confidential. Parenchymal hematoma (PH) was defined as hemorrhage with mass effect according to previously published criteria [9, 10]. sICH was defined as any hemorrhage in the brain on CT scan accompanied by neurological deterioration [9, 10].

### Statistical analysis

Characteristics of patients with and without intracranial hemorrhage (PH or sICH) were compared using the Chi-squared test, Fisher exact test, or Mann-Whitney U test. Multivariate analysis of variance regression models was used to assess the relationship between eosinophil level and sICH. Eosinophil level was included in multivariate analysis either as a continuous variable (eosinophil count) or as a categorical variable (high or low eosinophil count according to the median eosinophil count: 0*10^9^/L). We first included age and female sex in the model (model 1). Subsequently, variables were included in model 2 if they were associated with sICH (*P* < 0.10) or if they changed the estimated effect of eosinophils on sICH by more than 10% [1, 11, 12]. Generalized additive models (GAM) and dichotomous linear regression models were used to identify nonlinear relationships and to calculate the threshold effect of eosinophilia on sICH. Likelihood ratio tests were used to assess the modifying and interactive effects of eosinophils and subgroup variables on sICH. Finally, after controlling for potential confounders, mediation analysis was used to assess whether sICH mediated the relationship between eosinophilia and functional outcome. In addition, we calculated the direct, indirect, and overall effects of predictors on functional outcome through mediating variables (sICH) [1, 13]. All statistical analyses were performed using EmpowerStats (http://www.empowerstats.com, X&Y Solutions, Inc., Boston, MA). Two-tailed *P* values < 0.05 were considered statistically significant [1, 11, 12].

## Results

### Baseline characteristics of patients

A total of 328 patients with AIS (median age: 68 years) who underwent mechanical thrombectomy were included in this study. The main baseline characteristics of the patients according to the presence or absence of PH or sICH are summarized in Table 1. Patients with sICH had significantly higher NIHSS scores, more extensive ischemic lesions, more ICA occlusions, and bad collaterals. Eosinophil levels in the PH/sICH group were significantly lower than those in the no PH/sICH group (*P* < 0.001).

**Table 1:** Baseline characteristics of study population according to the presence/absence of PH or sICH

Characteristics	No PH (n = 250)	PH (n = 78)	*P*	No sICH (n = 270)	sICH (n = 58)	*P*
Age, y; median (IQR)	68.00 (56.00–76.00)	67.00 (58.50–74.00)	0.897	67.00 (56.00–75.00)	70.00 (62.25–75.00)	0.082
Female, n (%)	100 (40.00%)	43 (55.13%)	0.019	114 (42.22%)	29 (50.00%)	0.278
Atrial fibrillation, n (%)	100 (40.00%)	47 (60.26%)	0.002	114 (42.22%)	33 (56.90%)	0.041
Hypertension, n (%)	170 (68.00%)	55 (70.51%)	0.676	180 (66.67%)	45 (77.59%)	0.104
Diabetes, n (%)	45 (18.00%)	16 (20.51%)	0.619	50 (18.52%)	11 (18.97%)	0.937
Hyperlipidemia, n (%)	88 (35.20%)	27 (34.62%)	0.925	96 (35.56%)	19 (32.76%)	0.685
History of stroke, n (%)	36 (14.40%)	17 (21.79%)	0.121	40 (14.81%)	13 (22.41%)	0.154
Smoking, n (%)	77 (30.80%)	23 (29.49%)	0.826	85 (31.48%)	15 (25.86%)	0.399
Drinking, n (%)	58 (23.20%)	15 (19.23%)	0.462	62 (22.96%)	11 (18.97%)	0.507
Baseline NIHSS, median (IQR)	15.00 (12.00–18.00)	18.50 (16.00–23.00)	< 0.001	15.00 (12.00–19.00)	19.00 (16.00–23.75)	< 0.001
ASPECTS, median (IQR)	7.00 (7.00–8.00)	7.00 (6.00–7.00)	0.009	7.00 (7.00–8.00)	7.00 (6.00–7.00)	0.003
Occluded artery, n (%)			0.074			0.002
ICA	46 (18.40%)	24 (30.77%)		47 (17.41%)	23 (39.66%)	
M1 of the MCA	154 (61.60%)	45 (57.69%)		172 (63.70%)	27 (46.55%)	
Posterior circulation	32 (12.80%)	5 (6.41%)		31 (11.48%)	6 (10.34%)	
Others	18 (7.20%)	4 (5.13%)		20 (7.41%)	2 (3.45%)	
IVT, n (%)	78 (31.20%)	25 (32.05%)	0.888	85 (31.48%)	18 (31.03%)	0.947
Premorbid mRS, median (IQR)	0.00 (0.00–0.00)	0.00 (0.00–0.00)	0.321	0.00 (0.00–0.00)	0.00 (0.00–0.00)	0.425
Stroke etiology, n (%)			0.028			0.106
LAA	119 (47.60%)	24 (30.77%)		124 (45.93%)	19 (32.76%)	
Cardioembolic	118 (47.20%)	50 (64.10%)		131 (48.52%)	37 (63.79%)	
Others	13 (5.20%)	4 (5.13%)		15 (5.56%)	2 (3.45%)	
Collateral score, median (IQR)	0.00 (0.00–2.00)	0.00 (0.00–1.00)	0.183	0.00 (0.00–2.00)	0.00 (0.00–1.00)	0.015
OTR, median (IQR), min	343.00 (277.75–440.00)	337.00 (271.00–414.00)	0.475	344.50 (278.75–440.25)	340.50 (267.00–407.50)	0.316
Number of passes, median (IQR)	2.00 (1.00–3.00)	2.00 (1.00–3.00)	0.015	2.00 (1.00–3.00)	2.00 (1.00–3.00)	0.068
mTICI score 2b or 3, n (%)	217 (86.80%)	72 (92.31%)	0.190	238 (88.15%)	51 (87.93%)	0.963
Eosinophils, 10^9^/l; median (IQR)	0.01 (0.00–0.04)	0.00 (0.00–0.02)	< 0.001	0.01 (0.00–0.04)	0.00 (0.00–0.00)	< 0.001
mRS score	3.00 (1.00–4.00)	6.00 (4.00–6.00)	< 0.001	3.00 (1.00–4.00)	6.00 (5.00–6.00)	< 0.001
Poor outcome	141 (56.40%)	70 (89.74%)	< 0.001	156 (57.78%)	55 (94.83%)	< 0.001

ASPECTS, Alberta Stroke Program Early CT Score; IQR, interquartile range; ICA, internal carotid artery; IVT, intravenous thrombolysis; MCA, middle cerebral artery; mRS, modified Rankin Scale; mTICI, modified Thrombolysis in Cerebral Infarction; NIHSS, National Institutes of Health Stroke Scale; OTR, onset to reperfusion time; Posterior circulation, including basilar artery and intracranial part of the vertebral artery

### Univariate and multivariate analysis

On univariate analysis, AF, baseline NIHSS, ASPECTS, occluded artery, premorbid mRS, and collateral scores showed a positive association with sICH, whereas ASPECTS, other stroke etiologies, and collateral scores showed a negative association with sICH. Univariate analysis results for PH were similar to those for sICH.

Table 2 summarizes the results of multivariate regression analysis. Eosinophil count, when used as a continuous variable, showed an independent association with sICH with an adjusted odds ratio (OR) of 0.00 (95% confidence interval [CI], 0.00–0.00; *P* = 0.0012) after adjusting for age and female sex (model 1) and 0.00 (95% CI, 0.00–0.01; *P* = 0.0141) after adjusting for all potential covariates (model 2). For sensitivity analysis, we used eosinophil count as a categorical variable by dichotomization; the OR (95% CI) for sICH was 0.22 (0.11–0.46) for participants with higher eosinophil count compared with patients with lower eosinophil count. For the test of binary logistic regression of PH, a similar association was found between eosinophils and PH. Higher eosinophilia (OR 0.36, 95% CI: 0.19–0.67; *P* = 0.0013) remained an independent predictor of decreased risk of PH after adjusting for all potential covariates (model 2).

**Table 2:** Relationship between eosinophils and PH/sICH among patients with acute ischemic stroke in different models

	PH OR; (95% CI); *P* Value				sICH OR (95% CI)		
	Non-adjusted model	Model 1	Model 2		Non-adjusted model	Model 1	Model 2
Eosinophils	0.00; (0.00, 0.00); 0.0018	0.00; (0.00, 0.00); 0.0027	0.00; (0.00, 0.05); 0.0174		0.00; (0.00, 0.00); 0.0008	0.00; (0.00, 0.00); 0.0012	0.00; (0.00, 0.01); 0.0141
Eosinophils (dichotomous)	0.32; (0.19, 0.54); <0.0001	0.33; (0.19, 0.56); <0.0001	0.36; (0.19, 0.67); 0.0013		0.18; (0.09, 0.34); <0.0001	0.18; (0.10, 0.35); <0.0001	0.22; (0.11, 0.46); <0.0001

Non-adjusted model: not adjusted for other covariates

Model 1: adjusted for age and female sex

Model 2: adjusted for variables which were significantly associated with outcomes of interest (*P* < 0.10) or changed the estimates of eosinophils on outcomes of interest by more than 10% (Supplementary Tables 2–7 in the supplementary file)

CI, confidence interval; OR, odds ratio; PH, parenchymal hematoma; sICH, symptomatic intracranial hemorrhage

### Analyses of non-linear relationship

In the present study, we analyzed the nonlinear relationship between eosinophils and sICH/PH. No nonlinear relationship was found between eosinophils and sICH/PH (Supplementary Figs. 2–3). In addition, there were no statistically significant inflection points in the threshold effects analysis (*P* of 1 for the log-likelihood ratio test, suggesting that standard linear regression rather than dichotomous linear regression may be more appropriate for analyzing the relationship between eosinophils and sICH). Subgroup analysis further confirmed the association between eosinophils and sICH. As shown in Supplementary Tables 5, the interaction tests for age, AF, diabetes mellitus, and hyperlipidemia were statistically significant (*P* values for interactions < 0.05). Similar associations were found between eosinophilia and PH (Supplementary Table 5).

### Mediation analysis for functional outcome

**Fig. 1:**
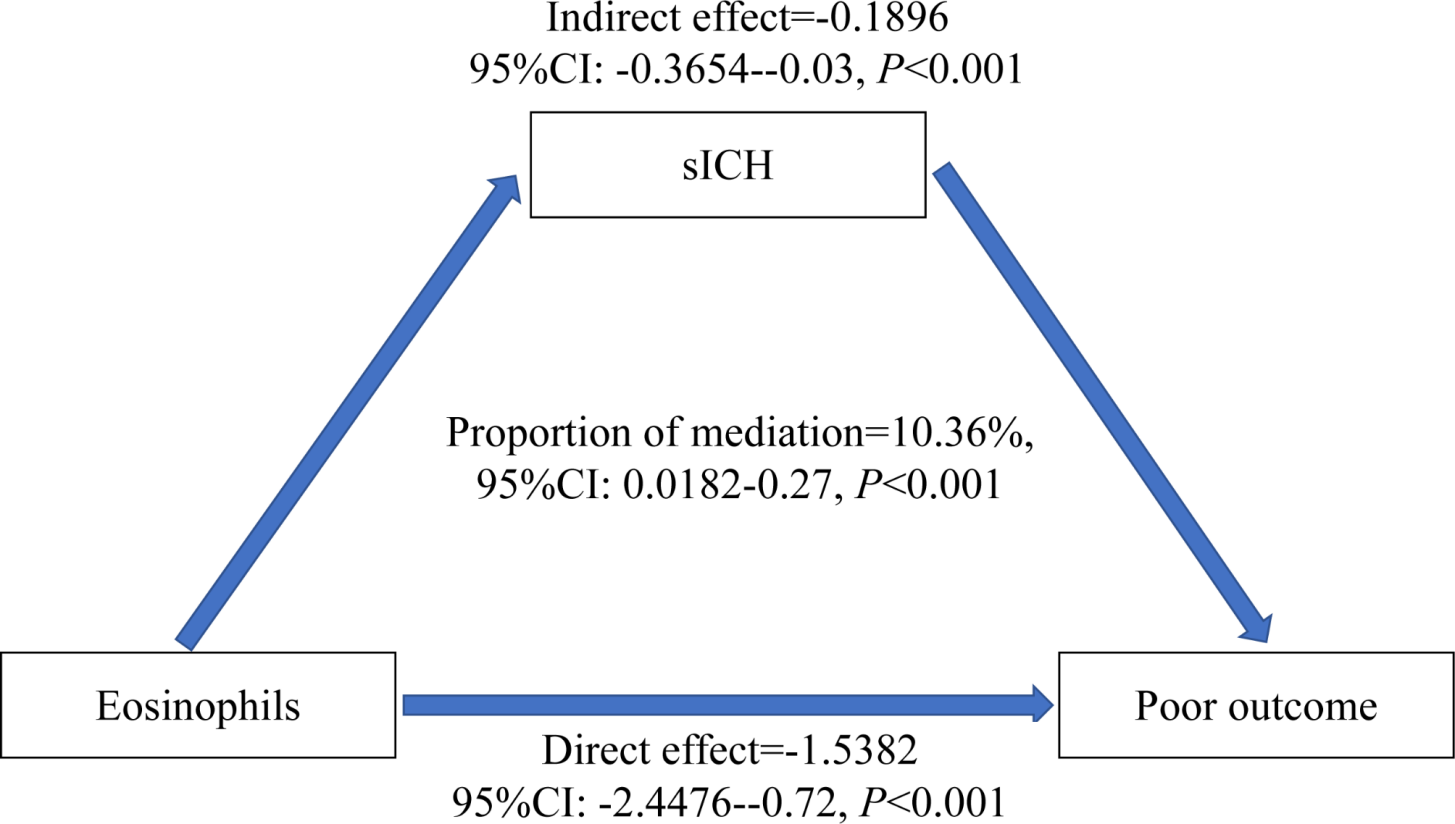
Schematic diagram of mediation analyses for functional outcome. The eosinophils were entered as predictors. sICH was entered as a mediator

Eosinophil count was also associated with poor outcome (as a continuous variable: OR, 0.00; 95% CI, 0.00–0.09; *P* = 0.0086; as a categorical variable: OR, 0.21; 95% CI, 0.10–0.46; *P* < 0.0001; Table 2 and Supplementary Table 3), which is consistent with previous studies [1, 14–17]. We then constructed a hypothetical model of the relationship between eosinophils, sICH, and functional outcome. Our results suggest that sICH partially mediates the relationship between eosinophils and poor outcomes (Fig. 1). The proportion of the total effect of eosinophils on poor outcomes mediated by sICH was 10.36% (95% CI, 1.82–27%). After removing the effects mediated by sICH, the direct effect of eosinophils on poor outcomes (total effect minus indirect effect) remained statistically significant (*P* < 0.001). However, no mediating effect of PH between eosinophilia and poor outcomes was observed in this study (Supplementary Fig. 4).

## Discussion

Endovascular therapy for AIS is clinically effective and offers cost savings compared with usual care alone. sICH is a serious complication after endovascular therapy for ischemic stroke, which increases the risk of death at 3 months and the likelihood of poor functional outcome [8, 16, 17]. The present study assessed the relationship between eosinophils, sICH, and functional outcome after mechanical thrombectomy in patients with AIS. Decreased eosinophil counts were associated with an increased risk of sICH/PH. In addition, there was an association between eosinophil count and functional outcome, which is consistent with previous studies [14–17]. More importantly, this study showed an important role of sICH in this association.

Many risk factors for sICH have been identified such as neutrophils and neutrophil-to-lymphocyte ratio [9, 18–21]. To the best of our knowledge, no study has investigated the effect of eosinophils on sICH after mechanical thrombectomy. In the present study, a decrease in eosinophils was associated with an increase in sICH. The mechanisms underlying these observations are not well established, but eosinophil-induced neuroprotection may play a critical regulatory role in the development of sICH. Eosinophils can secrete vascular endothelial growth factor and several chemokines [3–5]. IL-4 and IL-13 secreted by eosinophils can induce the activation of M2 phenotype microglia, which have a neuroprotective effect by promoting the resolution of inflammation. Furthermore, vascular endothelial growth factor may have neuroprotective effects by regulating angiogenesis [3–5]. Alternatively, a decrease in eosinophils may lead to reduced neuroprotection and more sICH, which in turn leads to a worse outcome.

Eosinophils are associated with both sICH and functional outcome. sICH partially mediates the relationship between decreased eosinophil levels and poor outcome. These data suggest that eosinophil-induced neuroprotection and subsequent complications of sICH may be one of the mechanisms underlying the association between eosinophils and functional outcome. Furthermore, we observed a significant association of eosinophils with poor outcome independent of sICH in multivariate analysis (mRS: β, -5.00; 95% CI, -8.71-1.30; *P* = 0.0086; poor outcome: OR, 0.00; 95% CI, 0.00–0.09; *P* = 0.0086. Supplementary Table 6). These results suggest an additional prognostic value of eosinophils when considering sICH. Thus, in addition to the sICH pathway, other pathways (e.g., stroke-associated pneumonia) may also contribute to poor outcome. In our previous study, lower eosinophil levels were associated with higher stroke-associated pneumonia, and stroke-associated pneumonia was found to play an important mediating role in the association of eosinophils with adverse outcomes. Given the above, it is possible that a decrease in eosinophils could lead to a higher risk of sICH and SAP, which in turn could lead to poor outcomes [1]. This hypothesis needs to be tested in animal studies.

Post-stroke reduction in eosinophil count results either due to a decrease in generation or an increase in death. Eosinophils mainly develop from CD34 + progenitor cells in the bone marrow, and the first step in their development is the expression of the IL-5 receptor α chain on the surface of CD34 + cells; administration of anti-IL-5 therapy significantly reduces mature eosinophils, early and late granulocytes in the bone marrow [22, 23]. More importantly, recent studies suggest that IL-5 expression in peripheral blood is reduced after stroke, which is independently associated with stroke severity and poor outcome [24, 25]; this suggests that reduced IL-5 may also be involved in the post-stroke reduction in eosinophil count. In addition, recent animal studies and clinical studies have shown an increase in TNF-α levels in peripheral blood after stroke; further, in vitro cellular assays have shown that exogenous TNF-α induces a decrease in eosinophil numbers [26, 27]. TNF-α may induce eosinophil death through the classical TNFR1/RIPK1/FADD/caspase-8 apoptotic signaling pathway and the TNFR1/RIPK1/RIPK3/MLKL programmed necrosis signaling pathway [26, 27].

Although eosinophilia was also associated with PH (OR, 0.00; 95% CI, 0.00–0.05; *P* = 0.0174), no mediation effect of PH in the association between eosinophilia and poor outcome was found in the present study. This discrepancy may be attributable to the small sample size, which weakens the statistical strength of the analysis. Another reason for the mediating effect of sICH rather than PH may be that poor outcome itself is a clinical definition. sICH reflects the degree of brain injury and clinical symptoms, whereas PH reflects only the degree of brain injury, which leads to a situation wherein sICH better reflects functional outcome. In addition, eosinophils appeared to be more sensitive to sICH (OR, 0.00; 95% CI, 0.00–0.01; *P* = 0.0141) or sICH to poor outcome (OR, 9.06; 95% CI, 2.31–35.48; *P* = 0. 0015) than eosinophils to PH (OR, 0.00; 95% CI, 0.00–0.05; *P* = 0.0174) or the effect size of PH on poor outcomes (OR, 6.27; 95% CI, 2.39–16.46; *P* = 0.0002). Further studies with a larger sample of patients with AIS are required to explore whether PH mediates the association between eosinophilia and poor outcomes.

The main strength of our study is that this was a comprehensive study with the established model to assess the effect of sICH on the relationship between eosinophil and functional outcomes. However, some limitations of this study should be acknowledged. First, eosinophil levels were measured at only a single time-point, and therefore, the results may vary depending on the possible rapid changes in their values after the onset of symptoms [9, 28]. Second, we neither explored the mechanisms by which eosinophils affect immunosuppressive and neuroprotective pathways nor investigated the factors which regulate changes in eosinophils after ischemic stroke in animal studies. These will be the focus of our next work, especially to explore the role of eosinophils in ischemic stroke and their mechanisms. Third, this was a retrospective single-center study with a small sample size. In addition, our cohort represents a subgroup of stroke patients who underwent thrombectomy; therefore, our findings may not be generalizable to the entire stroke patient population and further studies from other samples of AIS patients are required to validate our results. Fourth, the cross-sectional design of our study does not permit causal inferences. To compensate for this limitation, we performed a causal mediation analysis which suggested a potential association between eosinophilia, sICH, and functional outcome (Fig. 1; Supplementary Figs. 4–6). However, it is noteworthy that this is the first study to show a complex relationship between eosinophils, sICH, and functional outcome in patients with AIS experiencing MT. In this regard, attempts to maintain eosinophil counts have important implications for stroke prognosis, especially considering that clinical outcomes at 3 months remain unsatisfactory, with nearly half of all patients who are successfully reperfused presenting with an unfavorable functional prognosis [29].

## Conclusions

The present study showed that reduced eosinophil levels are associated with a high risk of sICH and poor outcome in patients with AIS experiencing MT, and that sICH is on the pathway of association between eosinophils and functional outcomes. However, PH was not found to mediate the association between eosinophils and poor outcome.

### Electronic supplementary material

Below is the link to the electronic supplementary material.

Supplementary Material 1

## Data Availability

The raw data supporting the conclusions of this article will be made available by the authors (Zhiliang Guo), without undue reservation.

## Abbreviations

AIS: Acute ischemic stroke
AF: Atrial fibrillation
ASPECTS: Alberta Stroke Program Early CT Score
CI: Confidence interval
IQR: Interquartile range
IVT: Intravenous thrombolysis
mRS: Modified Rankin Scale
MT: Mechanical thrombectomy
mTICI: Modified Thrombolysis in Cerebral Infarction
NIHSS: National Institutes of Health Stroke Scale
OR: Odds ratio
PH: Parenchymal hematoma
SAP: Stroke-associated pneumonia
sICH: Symptomatic intracranial hemorrhage

## References

[1] Guo Z, Hou J, Yu S, Zhang H, Yu S, Wang H (2022). Eosinophils, Stroke-Associated Pneumonia, and Outcome after Mechanical Thrombectomy for Acute ischemic stroke. Front Aging Neurosci.

[2] Yu S, Huang ZC, Wang HS, Liu SW, You SJ, Hou J, Guo ZL, Xiao GD (2023). Eosinophil: a new circulating biomarker for risk of poor outcome in stroke patients undergoing mechanical thrombectomy. Clin Interv Aging.

[3] Yu S, Luo Y, Zhang T, Huang C, Fu Y, Zhang Q (2021). Eosinophil-to-monocyte ratio is a potential biomarker in the prediction of functional outcome among patients with acute ischemic stroke. BMC Neurosci.

[4] Zierath D, Tanzi P, Shibata D, Becker KJ (2018). Cortisol is more important than Metanephrines in driving changes in Leukocyte Counts after Stroke. J Stroke Cerebrovasc Diseases: Official J Natl Stroke Association.

[5] Davoine F, Lacy P (2014). Eosinophil cytokines, chemokines, and growth factors: emerging roles in immunity. Front Immunol.

[6] Wang Y, Zhao X, Liu L, Soo YO, Pu Y, Pan Y (2014). Prevalence and outcomes of symptomatic intracranial large artery stenoses and occlusions in China: the chinese intracranial atherosclerosis (CICAS) study. Stroke.

[7] Xiao L, Ma M, Gu M, Han Y, Wang H, Zi W (2020). Renal impairment on clinical outcomes following endovascular recanalization. Neurology.

[8] Higashida RT, Furlan AJ, Roberts H, Tomsick T, Connors B, Barr J (2003). Trial design and reporting standards for intra-arterial cerebral thrombolysis for acute ischemic stroke. Stroke.

[9] Guo Z, Yu S, Xiao L, Chen X, Ye R, Zheng P (2016). Dynamic change of neutrophil to lymphocyte ratio and hemorrhagic transformation after thrombolysis in stroke. J Neuroinflamm.

[10] Hacke W, Kaste M, Fieschi C, von Kummer R, Davalos A, Meier D (1998). Randomised double-blind placebo-controlled trial of thrombolytic therapy with intravenous alteplase in acute ischaemic stroke (ECASS II). Second European-Australasian Acute Stroke Study Investigators. Lancet (London England).

[11] Guo Z, Xu G, Wang R, Hou J, Yu S, Wang H (2022). Free thyroxine, brain frailty and clock drawing test performance in patients with acute minor stroke or transient ischaemic attack. Clin Endocrinol.

[12] Hu S, Lan T, Wang S, Su L, Zou S, Ye J (2022). Serum chloride level is Associated with Abdominal aortic calcification. Front Cardiovasc Med.

[13] VanderWeele TJ (2016). Mediation analysis: a practitioner’s guide. Annu Rev Public Health.

[14] Zhao HM, Qin WQ, Wang PJ, Wen ZM (2019). Eosinopenia is a predictive factor for the severity of acute ischemic stroke. Neural Regeneration Research.

[15] Wang J, Ma L, Lin T, Li SJ, Chen LL, Wang DZ (2017). The significance of eosinophils in predicting the severity of acute ischemic stroke. Oncotarget.

[16] Semerano A, Strambo D, Martino G, Comi G, Filippi M, Roveri L (2020). Leukocyte Counts and Ratios are Predictive of Stroke Outcome and Hemorrhagic Complications independently of infections. Front Neurol.

[17] Cai H, Huang H, Yang C, Ren J, Wang J, Gao B, Pan W, Sun F, Zhou X, Zeng T, Hu J, Chen Y, Zhang S, Chen G (2021). Eosinophil-to-neutrophil ratio predicts poor prognosis of Acute ischemic stroke patients treated with intravenous thrombolysis. Front Neurol.

[18] van Kranendonk KR, Treurniet KM, Boers AMM, Berkhemer OA, van den Berg LA, Chalos V (2019). Hemorrhagic transformation is associated with poor functional outcome in patients with acute ischemic stroke due to a large vessel occlusion. J Neurointerventional Surg.

[19] van der Steen W, van der Ende NAM, van Kranendonk KR, Chalos V, van Oostenbrugge RJ, van Zwam WH et al. (2022). Determinants of symptomatic intracranial hemorrhage after endovascular stroke treatment: a retrospective cohort study. Stroke, 101161TROKEAHA121036195. Advance online publication. 10.1161/STROKEAHA.121.036195. 10.1161/STROKEAHA.121.036195 PMC9389940 35674042

[20] Duan Z, Wang H, Wang Z, Hao Y, Zi W, Yang D (2018). Neutrophil-lymphocyte ratio predicts Functional and Safety Outcomes after Endovascular Treatment for Acute ischemic stroke. Cerebrovasc Dis.

[21] Hao Y, Yang D, Wang H, Zi W, Zhang M, Geng Y (2017). Predictors for symptomatic intracranial hemorrhage after endovascular treatment of Acute ischemic stroke. Stroke.

[22] Menzies-Gow A, Flood-Page P, Sehmi R, Burman J, Hamid Q, Robinson DS, Kay AB, Denburg J (2003). Anti-IL-5 (mepolizumab) therapy induces bone marrow eosinophil maturational arrest and decreases eosinophil progenitors in the bronchial mucosa of atopic asthmatics. J Allergy Clin Immunol.

[23] Nagase H, Ueki S, Fujieda S (2020). The roles of IL-5 and anti-IL-5 treatment in eosinophilic diseases: Asthma, eosinophilic granulomatosis with polyangiitis, and eosinophilic chronic rhinosinusitis. Allergology International: Official Journal of the Japanese Society of Allergology.

[24] Coveney S, Murphy S, Belton O, Cassidy T, Crowe M, Dolan E, de Gaetano M, Harbison J, Horgan G, Marnane M, McCabe JJ, Merwick A, Noone I, Williams D, Kelly PJ (2022). Inflammatory cytokines, high-sensitivity C-reactive protein, and risk of one-year vascular events, death, and poor functional outcome after stroke and transient ischemic attack. Int J Stroke: Official J Int Stroke Soc.

[25] Li X, Lin S, Chen X, Huang W, Li Q, Zhang H, Chen X, Yang S, Jin K, Shao B (2019). The Prognostic Value of serum cytokines in patients with Acute ischemic stroke. Aging and Disease.

[26] Zhou H, Zhou M, Hu Y, Limpanon Y, Ma Y, Huang P, Dekumyoy P, Maleewong W, Lv Z (2022). TNF-α triggers RIP1/FADD/Caspase-8-Mediated apoptosis of astrocytes and RIP3/MLKL-Mediated necroptosis of neurons Induced by Angiostrongylus cantonensis infection. Cell Mol Neurobiol.

[27] Gordy C, Liang J, Pua H, He YW (2014). c-FLIP protects eosinophils from TNF-α-mediated cell death in vivo. PLoS ONE.

[28] Yang D, Huang H, Weng Y, Ren J, Yang C, Wang J (2021). Dynamic decrease in Eosinophil after Intravenous Thrombolysis predicts poor prognosis of Acute ischemic stroke: a longitudinal study. Front Immunol.

[29] Goyal M, Menon BK, van Zwam WH, Dippel DW, Mitchell PJ, Demchuk AM (2016).

