# Supplementary material for: Symptomatic intracranial hemorrhage mediates the association between eosinophils and 90-day outcomes after mechanical thrombectomy for acute ischemic stroke
Source: BMC Neurosci. 2023 Dec 8;24:64. doi: 10.1186/s12868-023-00820-5 (PMC10709893; doi:10.1186/s12868-023-00820-5)
Supplement: Supplementary file 1 — Supplementary Material 1 [file 12868_2023_820_MOESM1_ESM.doc]

**The supplementary file**

Symptomatic intracranial hemorrhage mediates the association between eosinophils and 90-day outcomes after mechanical thrombectomy for acute ischemic stroke

**Supplementary Table 1**. The selected covariates when eosinophils as a continuous variable

| Y | X | The selected covariates（Criterion 1） | The selected covariates（Criterion 2） |
| --- | --- | --- | --- |
| sICH | Eosinophils | Baseline NIHSS, Occluded artery | Age, Atrial fibrillatio, Baseline NIHSS, ASPECTS, Occluded artery, Premorbid mRS, Stroke etiology, Collateral score, Number of passes |
| PH | Eosinophils | Baseline NIHSS | Female, Atrial fibrillatio, Baseline NIHSS, ASPECTS, Occluded artery, Premorbid mRS, Stroke etiology, Collateral score, Number of passes |
| mRS score | Eosinophils | Baseline NIHSS, ASPECTS, Collateral score | Age, Female, Atrial fibrillation, Hypertension, Diabetes, History of stroke, Smoking, Drinking, Baseline NIHSS, ASPECTS, Occluded artery, Collateral score, OTR, mTICI score 2b or 3 |
| Poor outcome | Eosinophils | Baseline NIHSS | Age, Female, Atrial fibrillation, Hypertension, Diabetes, History of stroke, Smoking, Drinking, Baseline NIHSS, ASPECTS, Occluded artery, Collateral score, Number of passes, mTICI score 2b or 3 |

Criterion 1: These confounders changed the estimates of eosinophils on the outcomes of interest by more than 10% when introduce covariates into the basic model or remove covariates from the complete model.

Criterion 2: These variables were significantly associated with the outcomes of interest (*P* < 0.10) or changed the estimates of eosinophils on the outcomes of interest by more than 10%.

PH, parenchymal hematoma; sICH, symptomatic intracranial hemorrhage.

**Supplementary Table 2.** The selected covariates when eosinophils as a categorical variable (dichotomous)

| Y | X | The selected covariates（Criterion 1） | The selected covariates（Criterion 2） |
| --- | --- | --- | --- |
| sICH | Eosinophils (dichotomous) |  | Age, Atrial fibrillatio, Baseline NIHSS, ASPECTS, Occluded artery, Premorbid mRS, Stroke etiology, Collateral score |
| PH | Eosinophils (dichotomous) | Baseline NIHSS | Female, Atrial fibrillatio, Baseline NIHSS, ASPECTS, Occluded artery, Premorbid mRS, Stroke etiology, Collateral score |
| mRS score | Eosinophils (dichotomous) | Baseline NIHSS, ASPECTS, Dysphagia, Collateral score | Age, Female, Atrial fibrillation, Hypertension, Diabetes, History of stroke, Smoking, Drinking, Baseline NIHSS, ASPECTS, Occluded artery, Dysphagia, Collateral score, OTR, mTICI score 2b or 3 |
| Poor outcome | Eosinophils (dichotomous) | Baseline NIHSS | Age, Female, Atrial fibrillation, Hypertension, Diabetes, History of stroke, Smoking, Drinking, Baseline NIHSS, ASPECTS, Occluded artery, Dysphagia, Collateral score, Number of passes, mTICI score 2b or 3 |

Criterion 1: These confounders changed the estimates of eosinophils on the outcomes of interest by more than 10% when introduce covariates into the basic model or remove covariates from the complete model.

Criterion 2: These variables were significantly associated with symptomatic intracranial hemorrhage score (*P* < 0.10) or changed the estimates of eosinophils on the outcomes of interest by more than 10%.

PH, parenchymal hematoma; sICH, symptomatic intracranial hemorrhage.

**Supplementary Table 3.** Relationship between eosinophils and mRS/poor outcome among patients with acute ischemic stroke in different models

|  | mRS  β; (95% CI); *P* Value | | |  | Poor outcome (mRS score 3-6)  OR; (95% CI); *P* Value | | |
| --- | --- | --- | --- | --- | --- | --- | --- |
|  | Non-adjusted model | Model 1 | Model 2 |  | Non-adjusted model | Model 1 | Model 2 |
| Eosinophils | -12.43 (-17.26, -7.60) <0.0001 | -11.30 (-15.89, -6.71) <0.0001 | -5.36（-9.24，-1.48） 0.0072 |  | 0.00 (0.00, 0.00) <0.0001 | 0.00 (0.00, 0.00) 0.0001 | 0.00（0.00，0.14） 0.0124 |
| Eosinophils (dichotomous) | -1.63 (-2.04, -1.22) <0.0001 | -1.51 (-1.91, -1.12) <0.0001 | -0.80（-1.15，-0.44） <0.0001 |  | 0.18 (0.10, 0.31) <0.0001 | 0.17 (0.10, 0.30) <0.0001 | 0.20（0.09，0.45） 0.0001 |

Non-adjusted model: we did not adjust other covariates.

Model 1: we adjusted age and female.

Model 2: we adjusted variables which were significantly associated with outcomes of interest (*P* < 0.10) or changed the estimates of eosinophils on outcomes of interest by more than 10% (supplementary Table 2-7 in the supplementary file).

CI indicates confidence interval; OR, odds ratio; PH, parenchymal hematoma; sICH, symptomatic intracranial hemorrhage.

**Supplementary Table 4.** The results of threshold effect analysis

| Outcome: | sICH  OR; (95% CI); *P* Value | PH  OR; (95% CI); *P* Value |
| --- | --- | --- |
| Model I |  |  |
| A linear model | 0.00 (0.00, 0.01) 0.0141 | 0.00 (0.00, 0.05) 0.0174 |
| Model II(two-piecewise linear regression model) |  |  |
| Inflection point of eosinophils(K) | 0 | 0 |
| < K | NA | NA |
| > K | 0.00 (0.00, 0.01) 0.0141 | 0.00 (0.00, 0.05) 0.0174 |
| *P* for log likelihood ratio test | 1.000 | 1.000 |

Effect: eosinophils; Cause: PH/sICH; Adjusted: variables which were significantly associated with outcomes of interest (*P* < 0.10) or changed the estimates of eosinophils on outcomes of interest by more than 10% (supplementary Table 2-6 in the supplementary file). OR, odds ratio.

**Supplementary Table 5. Subgroup analyses on the association between eosinophils and hemorrhagic transformation among patients with acute ischemic stroke experiencing mechanical thrombectomy**

| Subgroup | N | Symptomatic intracranial hemorrhage | | | Parenchymal hematoma | | |
| --- | --- | --- | --- | --- | --- | --- | --- |
|  |  | OR (95%CI) | *P* value | *P* for interaction | OR (95%CI) | *P* value | *P* for interaction |
| Age, years |  |  |  | 0.0010 |  |  | 0.2779 |
| ＜65 | 135 | 0.00 (0.00, 0.00) | 0.0377 |  | 0.00 (0.00, 0.01) | 0.0224 |  |
| ≥65 | 193 | 0.00 (0.00, 2587.32) | 0.2878 |  | 0.00 (0.00, 253.27) | 0.2072 |  |
| Sex |  |  |  | 0.5019 |  |  | 0.1084 |
| Male | 185 | 0.00 (0.00, 2.50) | 0.0588 |  | 0.00 (0.00, 0.00) | 0.0051 |  |
| Female | 143 | 0.00 (0.00, 6040.88) | 0.1360 |  | 0.00 (0.00, 10049.93) 0.4536 | 0.4536 |  |
| Atrial fibrillation |  |  |  | 0.0007 |  |  | 0.0031 |
| No | 181 | 0.00 (0.00, 0.00) | 0.0088 |  | 0.00 (0.00, 0.00) | 0.0090 |  |
| Yes | 147 | .09 (0.00, inf.) | 0.8343 |  | 3.90 (0.00, inf.) | 0.8851 |  |
| Hypertension |  |  |  | 0.1057 |  |  | 0.7542 |
| No | 103 | 0.00 (0.00, 0.00) | 0.0304 |  | 0.00 (0.00, 0.96) | 0.0497 |  |
| Yes | 225 | 0.00 (0.00, 0.91) | 0.0488 |  | 0.00 (0.00, 0.90) | 0.0486 |  |
| Diabetes |  |  |  | 0.0389 |  |  | 0.3737 |
| No | 267 | 0.00 (0.00, 0.00) | 0.0091 |  | 0.00 (0.00, 0.01) | 0.0137 |  |
| Yes | 61 | Inf (0.00, Inf) | 0.9985 |  | Inf (Inf, Inf) | <0.0001 |  |
| Hyperlipidemia |  |  |  | 0.0444 |  |  | 0.5694 |
| No | 213 | 0.00 (0.00, 1940.00) | 0.2252 |  | 0.00 (0.00, 763.05) | 0.2151 |  |
| Yes | 115 | 0.00 (0.00, 0.00) | 0.0227 |  | 0.00 (0.00, 0.00) | 0.0094 |  |
| History of stroke |  |  |  | 0.1517 |  |  | 0.0597 |
| No | 275 | 0.00 (0.00, 0.37) | 0.0395 |  | 0.00 (0.00, 4.74) | 0.0811 |  |
| Yes | 53 | 0.00 (0.00, Inf) | 0.9980 |  | 0.00 (0.00, Inf) | 0.9995 |  |
| Smoking |  |  |  | 0.9389 |  |  | 0.1995 |
| No | 228 | 0.00 (0.00, 0.13) | 0.0345 |  | 0.00 (0.00, 20.75) | 0.1151 |  |
| Yes | 100 | 0.00 (0.00, 0.01) | 0.0418 |  | 0.00 (0.00, 0.00) | 0.0049 |  |
| Drinking |  |  |  | 0.6502 |  |  | 0.4855 |
| No | 255 | 0.00 (0.00, 0.00) | 0.0134 |  | 0.00 (0.00, 2.76) | 0.0672 |  |
| Yes | 73 | 0.00 (0.00, Inf) | 0.9992 |  | 0.00 (0.00, inf.) | 0.1733 |  |
| Baseline NIHSS |  |  |  | 0.2057 |  |  | 0.5111 |
| ＜16 | 151 | 0.00 (0.00, 371.49) | 0.1023 |  | 0.00 (0.00, 24960.78) | 0.2626 |  |
| ≥16 | 177 | 0.00 (0.00, 0.36) | 0.0444 |  | 0.00 (0.00, 20.63) | 0.0923 |  |
| IVT |  |  |  | 0.4804 |  |  | 0.8243 |
| No | 225 | 0.00 (0.00, 0.04) | 0.0300 |  | 0.00 (0.00, 38.93) | 0.1178 |  |
| Yes | 103 | 0.00 (0.00, 10666.09) | 0.1057 |  | 0.00 (0.00, 3.74) | 0.0626 |  |

In the multivariate models, confounding factors were included unless the variable was used as a stratification variable.

IVT, intravenous thrombolysis; N, No. of participants; NIHSS, National Institutes of Health Stroke Scale; OR, odds ratio.

**Supplementary Table 6.** Association of eosinophils with poor outcome was independent of sICH

| Exposure | Non-adjusted | Model 1 | Model 2 |
| --- | --- | --- | --- |
|  | β/OR; (95%CI); *P* Value | β/OR; (95%CI); *P* Value | β/OR; (95%CI); *P* Value |
| mRS |  |  |  |
| Eosinophils | -12.43 (-17.26, -7.60) <0.0001 | -11.30 (-15.89, -6.71) <0.0001 | -5.00 (-8.71, -1.30) 0.0086 |
| Eosinophils (dichotomous) |  |  |  |
| Low eosinophil level | 0 | 0 | 0 |
| High eosinophil level | -1.63 (-2.04, -1.22) <0.0001 | -1.51 (-1.91, -1.12) <0.0001 | -0.72 (-1.06, -0.38) <0.0001 |
| Poor outcome (mRS score 3-6) |  |  |  |
| Eosinophils | 0.00 (0.00, 0.00) <0.0001 | 0.00 (0.00, 0.00) 0.0001 | 0.00 (0.00, 0.09) 0.0086 |
| Eosinophils (dichotomous) |  |  |  |
| Low eosinophil level | 1.0 | 1.0 | 1.0 |
| High eosinophil level | 0.18 (0.10, 0.31) <0.0001 | 0.17 (0.10, 0.30) <0.0001 | 0.21 (0.10, 0.46) <0.0001 |

Non-adjusted model: we did not adjust other covariates.

Model 1: we adjusted age and sex.

Model 2: we adjusted variables which were significantly associated with outcomes of interest (*P* < 0.10) or changed the estimates of eosinophils on outcomes of interest by more than 10%.

mRS, modified Rankin Scale; OR, odds ratio; sICH, symptomatic intracranial hemorrhage.

**Supplementary Table 7.** Association of eosinophils with poor outcome was independent of PH

| Exposure | Non-adjusted | Model 1 | Model 2 |
| --- | --- | --- | --- |
|  | β/OR; (95%CI); *P* Value | β/OR; (95%CI); *P* Value | β/OR; (95%CI); *P* Value |
| mRS |  |  |  |
| Eosinophils | -12.43 (-17.26, -7.60) <0.0001 | -11.30 (-15.89, -6.71) <0.0001 | -5.20 (-8.95, -1.45) 0.0070 |
| Eosinophils (dichotomous) |  |  |  |
| Low eosinophil level | 0 | 0 | 0 |
| High eosinophil level | -1.63 (-2.04, -1.22) <0.0001 | -1.51 (-1.91, -1.12) <0.0001 | -0.78 (-1.12, -0.45) <0.0001 |
| Poor outcome (mRS score 3-6) |  |  |  |
| Eosinophils | 0.00 (0.00, 0.00) <0.0001 | 0.00 (0.00, 0.00) 0.0001 | 0.00 (0.00, 0.10) 0.0086 |
| Eosinophils (dichotomous) |  |  |  |
| Low eosinophil level | 1.0 | 1.0 | 1.0 |
| High eosinophil level | 0.18 (0.10, 0.31) <0.0001 | 0.17 (0.10, 0.30) <0.0001 | 0.21 (0.10, 0.44) <0.0001 |

Non-adjusted model: we did not adjust other covariates.

Model 1: we adjusted age and sex.

Model 2: we adjusted variables which were significantly associated with outcomes of interest (*P* < 0.10) or changed the estimates of eosinophils on outcomes of interest by more than 10%.

mRS, modified Rankin Scale; OR, odds ratio; PH, parenchymal hematoma.

**Supplementary Figure 1.** Flow chart of patient cohort


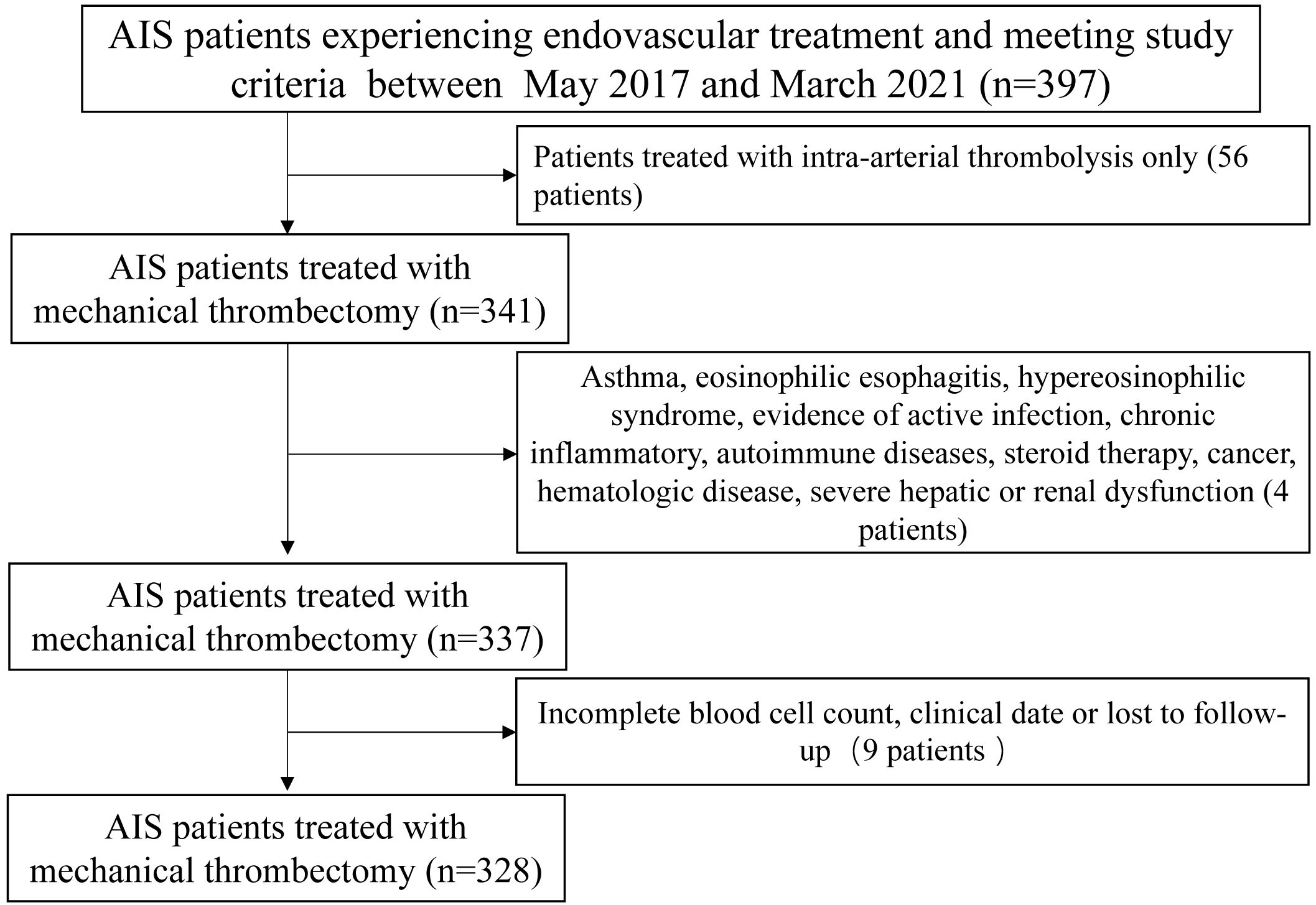


AIS: Acute ischemic stroke.

**Supplementary Figure 2.** The relationship between eosinophils and sICH in patients with acute ischemic stroke experiencing mechanical thrombectomy


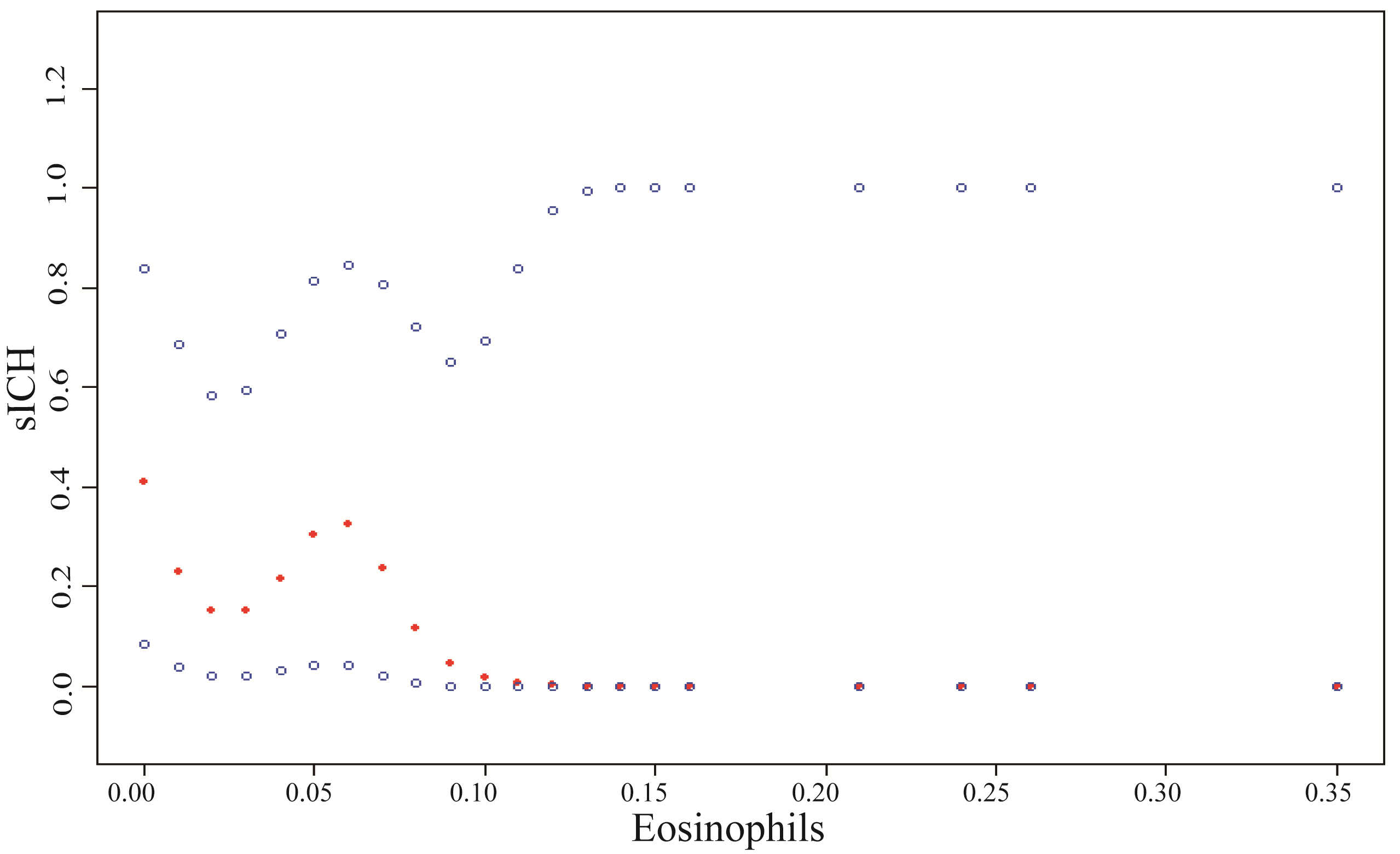


The red dots in the middle of the figure represent the smooth curve fit, and the blue dots represent the 95% confidence interval of the fit. The scatter plot of curve fit results mainly reflect the probability of sICH (Y-axis) with different eosinophil levels (X-axis). Adjusted for variables that were significantly associated with sICH (*P* < 0.10) or changed the estimates of eosinophils on sICH by more than 10% (supplementary Table 2-6 in the supplementary file).

sICH, symptomatic intracranial hemorrhage.

**Supplementary Figure 3.** The relationship between eosinophils and PH in patients with acute ischemic stroke experiencing mechanical thrombectomy


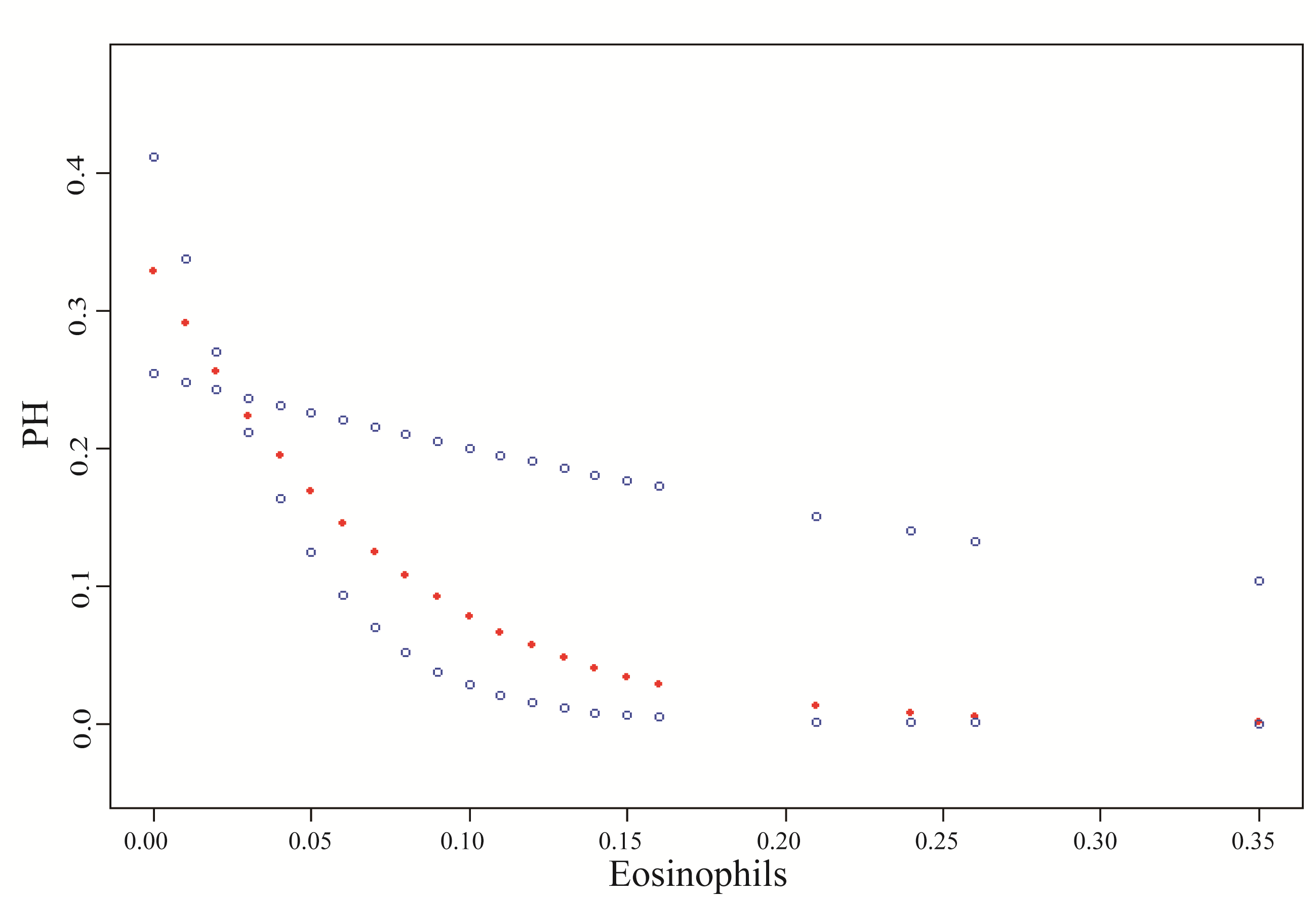


The red dots in the middle of the figure represent the smooth curve fit, and the blue dots represent the 95% confidence interval of the fit. The scatter plot of curve fit results mainly reflect the probability of PH (Y-axis) with different eosinophil levels (X-axis). Adjusted for variables that were significantly associated with PH (*P* < 0.10) or changed the estimates of eosinophils on PH by more than 10% (supplementary Table 2-6 in the supplementary file).

PH, parenchymal hematoma.

**Supplementary Figure 4.** Schematic diagram of mediation analyses for poor outcome


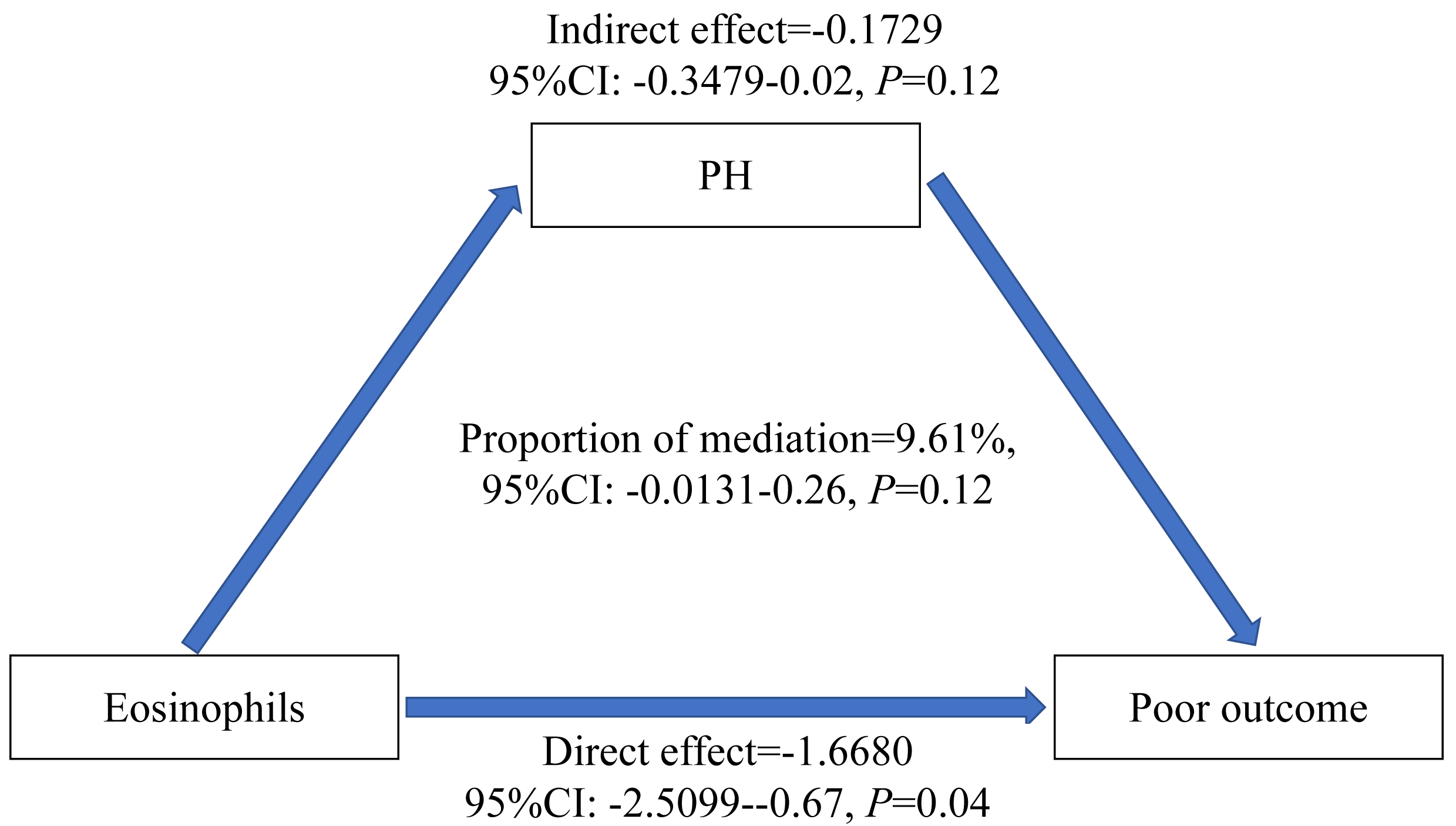


The eosinophils were entered as predictors. Parenchymal hematoma was entered as a mediator. The proportion of the total effect of eosinophils on the poor outcome mediated by parenchymal hematoma was 9.61% (95% CI, -1.31%–26%). The direct effect of eosinophils on the poor outcome (total effect minus indirect effect) was mot statistically significant (*P*=0.12) after removing the effect mediated by parenchymal hematoma.

PH, parenchymal hematoma.

**Supplementary Figure 5.** Schematic diagram of mediation analyses for mRS score


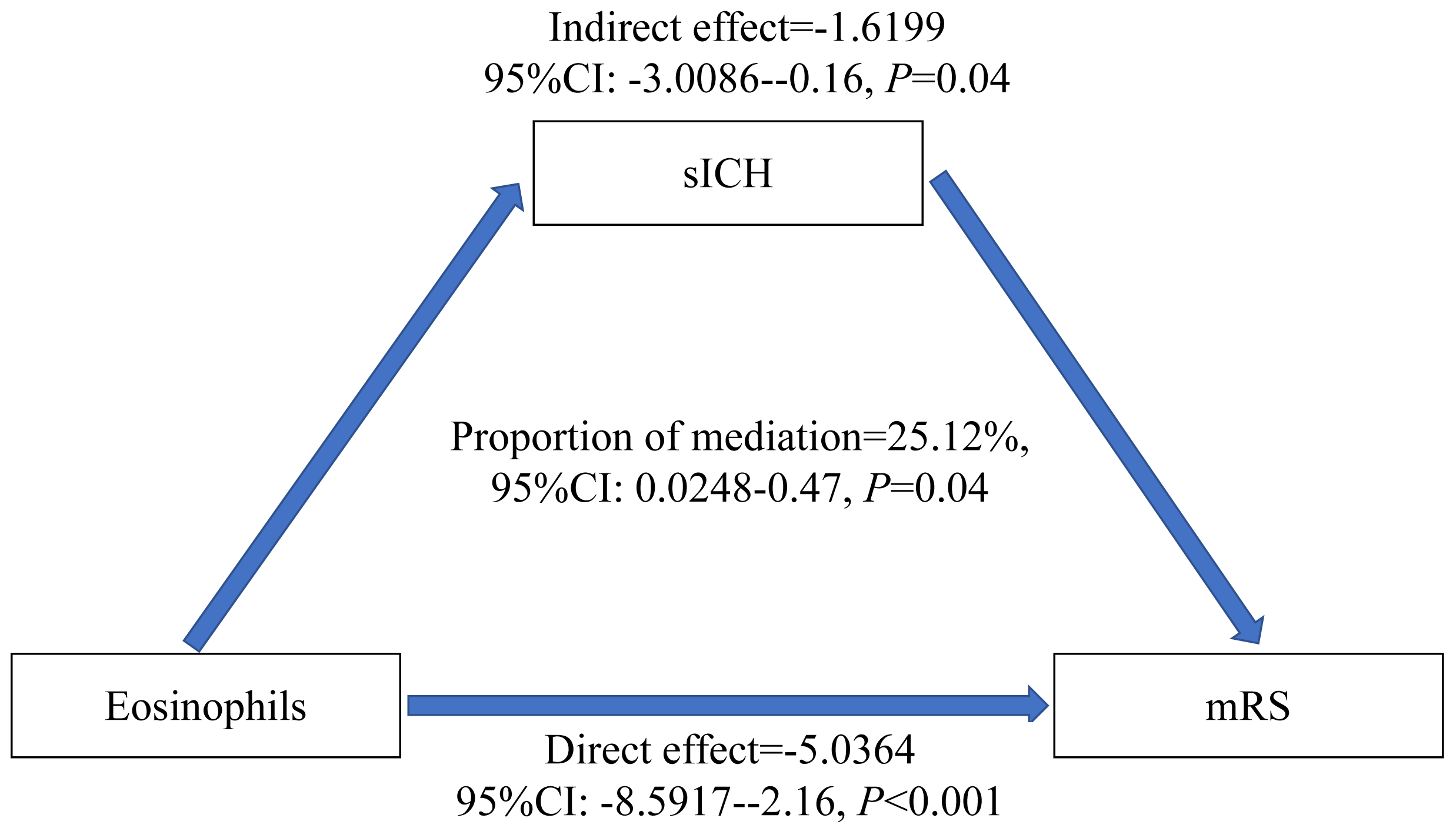


The eosinophils were entered as predictors. Symptomatic intracranial hemorrhage score was entered as a mediator. The proportion of the total effect of eosinophils on the mRS score mediated by symptomatic intracranial hemorrhage was 25.12% (95% CI, 2.48%–47%). The direct effect of eosinophils on the mRS score (total effect minus indirect effect) was still statistically significant (*P*=0.04) after removing the effect mediated by symptomatic intracranial hemorrhage.

mRS, modified Rankin Scale; sICH, symptomatic intracranial hemorrhage.

**Supplementary Figure 6.** Schematic diagram of mediation analyses for mRS score


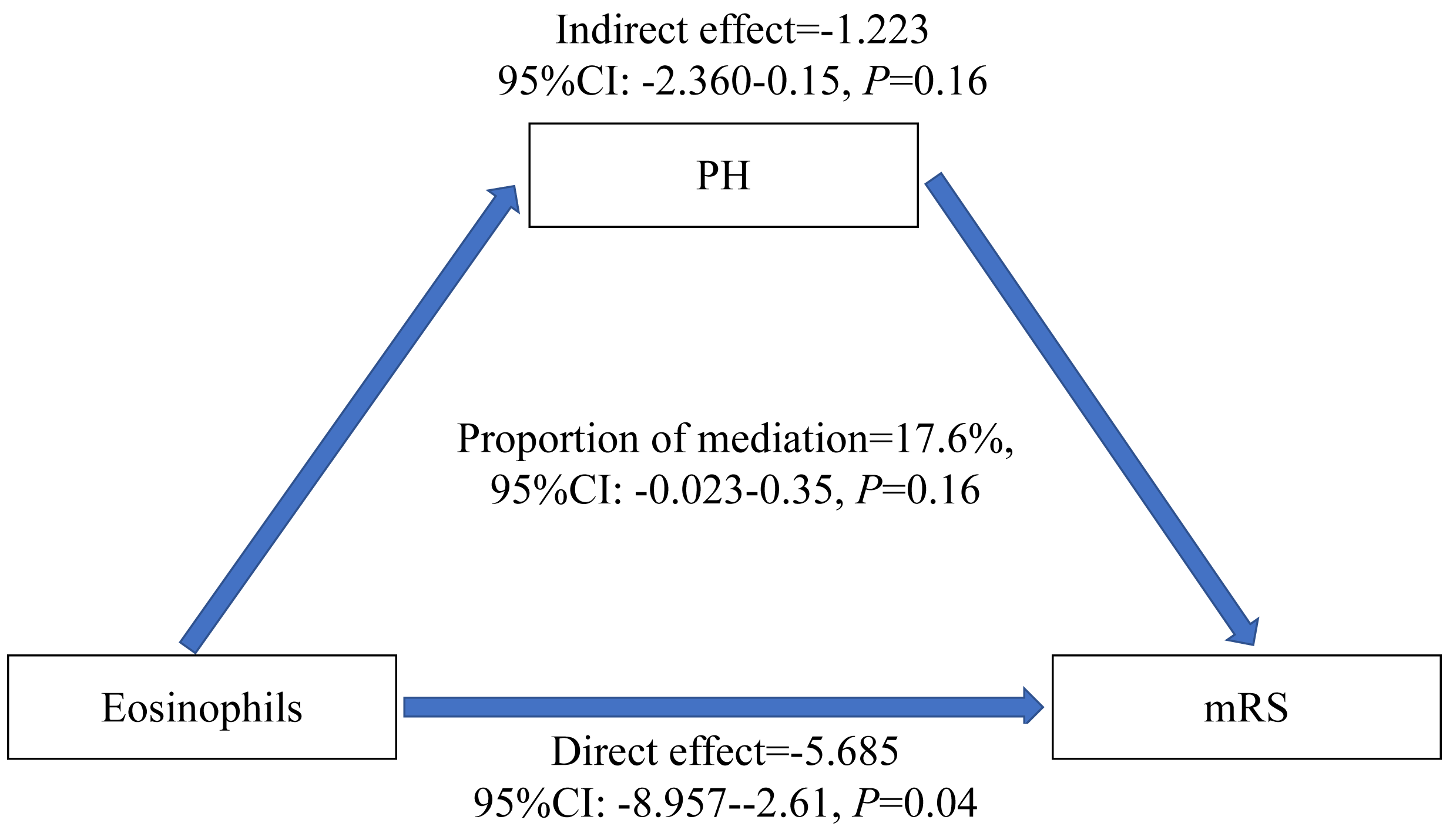


The eosinophils were entered as predictors. Parenchymal hematoma was entered as a mediator. The proportion of the total effect of eosinophils on the mRS score mediated by parenchymal hematoma was 17.6% (95% CI, -2.3%–35%). The direct effect of eosinophils on the poor outcome (total effect minus indirect effect) was mot statistically significant (*P*=0.16) after removing the effect mediated by parenchymal hematoma.

PH, parenchymal hematoma.
